# Identification of Low Molecular Weight Glutenin Alleles by Matrix-Assisted Laser Desorption/Ionization Time-Of-Flight Mass Spectrometry (MALDI-TOF-MS) in Common Wheat (*Triticum aestivum* L.)

**DOI:** 10.1371/journal.pone.0138981

**Published:** 2015-09-25

**Authors:** Aili Wang, Li Liu, Yanchun Peng, Shahidul Islam, Marie Applebee, Rudi Appels, Yueming Yan, Wujun Ma

**Affiliations:** 1 State Agriculture Biotechnology Centre, School of Veterinary & Life Sciences, Murdoch University, Australia Export Grains Innovation Centre (AEGIC), Perth, WA, 6150, Australia; 2 Key Laboratory of Genetics and Biotechnology, College of Life Science, Capital Normal University, Beijing, 100037, China; 3 National Wheat Improvement Centre, Institute of Crop Sciences, Chinese Academy of Agricultural Sciences (CAAS), No. 12 Zhongguancun South Street, Beijing, 100081, China; 4 College of Plant Science and Technology, Huazhong Agricultural University, Wuhan, 430070, Hubei, China; 5 South Australian Research & Development Institute, Waite Campus, 2b Hartley Grove, Urrbrae, SA, 5064, Australia; Saint Mary's University, CANADA

## Abstract

Low molecular weight glutenin subunits (LMW-GS) play an important role in determining dough properties and breadmaking quality. However, resolution of the currently used methodologies for analyzing LMW-GS is rather low which prevents an efficient use of genetic variations associated with these alleles in wheat breeding. The aim of the current study is to evaluate and develop a rapid, simple, and accurate method to differentiate LMW-GS alleles using matrix-assisted laser desorption/ionization time-of-flight mass spectrometry. A set of standard single LMW-GS allele lines as well as a suite of well documented wheat cultivars were collected from France, CIMMYT, and Canada. Method development and optimization were focused on protein extraction procedures and MALDI-TOF instrument settings to generate reproducible diagnostic spectrum peak profiles for each of the known wheat LMW-GS allele. Results revealed a total of 48 unique allele combinations among the studied genotypes. Characteristic MALDI-TOF peak patterns were obtained for 17 common LMW-GS alleles, including 5 (b, a or c, d, e, f), 7 (a, b, c, d or i, f, g, h) and 5 (a, b, c, d, f) patterns or alleles for the Glu-A3, Glu-B3, and Glu-D3 loci, respectively. In addition, some reproducible MALDI-TOF peak patterns were also obtained that did not match with any known alleles. The results demonstrated a high resolution and throughput nature of MALDI-TOF technology in analyzing LMW-GS alleles, which is suitable for application in wheat breeding programs in processing a large number of wheat lines with high accuracy in limited time. It also suggested that the variation of LMW-GS alleles is more abundant than what has been defined by the current nomenclature system that is mainly based on SDS-PAGE system. The MALDI-TOF technology is useful to differentiate these variations. An international joint effort may be needed to assign allele symbols to these newly identified alleles and determine their effects on end-product quality attributes.

## Introduction

Wheat seed storage proteins are composed of two major fractions, gliadins and glutenins. Based on their electrophoretic mobility, glutenin proteins are divided into high molecular weight glutenin subunits (HMW-GS) and low molecular weight glutenin subunits (LMW-GS.) [1]. About 20% of the whole glutenin fraction is HMW-GS and 80% is LMW-GS [2]. LMW-GS is highly polymorphic and mainly encoded by genes on complex loci Glu-A3, Glu-B3, and Glu-D3 on the short arms of group 1 chromosomes 1A, 1B, and 1D, respectively [3,4]. It possesses highly significant effects on dough physical properties especially dough extensibility, which is highly important for breadmaking [5–9]. Utilization of genetic variations associated with LMW-GS is currently an important task in modern wheat breeding.

In bread wheat cultivars, Gupta and Shepherd identified 20 different LMW-GS banding patterns by SDS-PAGE, six controlled by Glu-A3 (a, b, c, d, e, f), nine by Glu-B3 (a, b, c, d, e, f, g, h, i) and five by Glu-D3 (a, b, c, d, e) [3]. These banding patterns were then defined as LMW-GS alleles. The allele effect rankings for dough physical properties were also established, including Glu-A3: b>d>e>c; Glu-B3: i>b = a>e = f = g = h>c; Glu-D3: e>b>a>c>d. Ma et al. [6] demonstrated that selecting appropriate LMW-GS alleles is vital important in achieving balanced wheat dough physical properties.

Currently, two analytical systems are predominantly used for differentiating LMW-GS alleles, including sodium dodecyl sulfate polyacrylamide gel electrophoresis (SDS-PAGE) [10], and reversed-phase high-performance liquid chromatography (RP-HPLC) [11,12]. However, Glu-3 loci for LMW-GS consist of a multigene family of about 30–40 variable genes. The LMW-GS composition is highly polymorphic and often one allele is composed of multiple proteins; it is therefore often difficult to accurately identify and analyze the LMW-GS by the currently established methods due to a large number of expressed subunits and their overlapping mobilities with other proteins such as the abundant gliadin proteins. Due to the common scoring errors in determining LMW-GS compositions by the current analyzing methods [13,14], the LMW-GS variation on wheat quality is less utilized than these of HMW-GS in wheat breeding. A current large international collaborating effort is focused on refining the LMW-GS nomenclature system [15].

In recent years, new technologies such as two-dimensional electrophoresis (2-DE) and N-terminal amino acid sequences were developed to characterize and define LMW-GS [16], which have greatly improved the accuracy in identifying LMW-GS alleles and understanding their structures and functions. However, these technologies are of high cost and low throughput, not suitable for using in large scale wheat breeding programs that require accurately processing a large number of samples in a given short period. Matrix-assisted laser desorption/ionisation time-of-flight mass spectrometry (MALDI-TOF-MS) has been proven to be a powerful tool for wheat storage protein analysis [17,18]. It appears to be highly accurate and sensitive, only a small sample is required (normally less than 1 pmol), and is faster to perform (requiring about one minute per sample) comparing with other common separation methods [18,19]. The high throughput is particularly attractive for the possibility of rapid variety identification. It is most suitable for dealing with a large number of samples in a short time, ideal for wheat breeding programs, wheat grain trading, etc. Liu et al. [17] has successfully applied this technology in analyzing HMW-GS alleles and have identified a number of new alleles from old wheat varieties.

For the LMW-GS alleles, Muccilli et al. [20] analyzed the characteristics of the B- and C-type low molecular weight glutenin subunits by MALDI-TOF. However, allele specific MALDI-TOF profiles for LMW-GS alleles have not been established. MALDI-TOF-MS technology is still not efficiently used as an analytical procedure for wheat breeding. The aim of the current study is to use MALDI-TOF technology as a tool for rapid and accurately differentiating LMW-GS alleles in wheat breeding through establishing allele specific MALDI-TOF spectrum profiles.

## Materials and Methods

### 2.1 Wheat Material

A total of 60 hexaploid wheat lines with known LMW-GS compositions were used to establish characteristic MALDI-TOF peak pattern for each LMW-GS allele. Aroona and its 16 substitution lines with different Glu-3 alleles detected by protein mobility were sourced from South Australian Research & Development Institute Grain Quality Research Laboratory and were initially used to gain allele specific spectrum peak patterns (Table 1). A collection of 18 international reference varieties [21] and 25 hexaploid gene deletant lines with different Glu-3 alleles defined by SDS-PAGE were then used to verify the patterns obtained from the Aroona lines. The final allele patterns were put into use to analyze another 202 hexaploid wheat lines, including commercial cultivars and advanced breeding lines.

**Table 1:** Single *Glu-3* allele substitution lines of *Triticum aestivum* var. Aroona and their donor parents.

	Arils*		*Glu-A3*	*Glu-B3*	*Glu-D3*	Donor Parent
1	Aril 15–4	*A3a*	*a*	*b*	*c*	Chinese Spring
2	Aril 16–1	*A3b*	*b*	*b*	*c*	Gabo
3	Aroona	*A3c*	*c*	*b*	*c*	Aroona
4	Aril 18–5	*A3d*	*d*	*b*	*c*	Orca
5	Aril 19–2	*A3e*	*e*	*b*	*c*	Bungulla
6	Aril 20–1	*A3f*	*f*	*b*	*c*	BT2288A
7	Aril 21–2	*B3a*	*c*	*a*	*c*	Chinese Spring
8	Aroona	*B3b*	*c*	*b*	*c*	Aroona
9	Aril 23–4	*B3c*	*c*	*c*	*c*	Halberd
10	Aril 24–3	*B3d*	*c*	*d*	*c*	Orca
11	Aril 26–1	*B3f*	*c*	*f*	*c*	Gawain
12	Aril 27–6	*B3g*	*c*	*g*	*c*	Millewa
13	Aril 28–4	*B3h*	*c*	*h*	*c*	Sonalika
14	Aril 29–4	*B3i*	*c*	*i*	*c*	Jufy 1
15	Aril 30–1	*D3a*	*c*	*b*	*a*	Chinese Spring
16	Aril 36–2	*D3b*	*c*	*b*	*b*	Bungulla
17	Aroona	*D3c*	*c*	*b*	*c*	Aroona
18	Aril 33–1	*D3d*	*c*	*b*	*d*	Jufy 1
19	Aril 35–1	*D3f*	*c*	*b*	*f*	India 115

* Aril denotes Aroona recombinant inbred line (5 backcrosses)

Data Explorer Raw Data files for all lines in table 1 are included in S1 File.

### 2.2 LMW-GS Protein Extraction and Sample Preparation

#### 2.2.1 Protein extraction

Various methods of LMW-GS protein extraction were tested and optimized. The final method used is modified from Singh et al. [22] and Melas et al. [23]. About 15 mg of crushed seeds or flour were weighed followed by adding 1 ml 70% ethanol into the tube and vortexing for 30 min at room temperature. Centrifuge at 10,000 rpm for 5 min and then discard the supernatant. Add 1 ml of 55% iso-propanol into the pellet, mix well and put into 65°C water bath for 30 min. Centrifuge at 10,000 rpm for 5 min and discard the supernatant. The above steps were repeated twice. Supernatant must be discarded entirely at every step to thoroughly remove albumin, globulin and gliadin fractions. Add 150 μl of extraction buffer (50% iso-propanol, 80 mM Tris-HCl, pH8.0, and 1% DTT) at 65°C water bath for 30 min. After centrifugation, glutenin fractions were alkylated by adding an equal volume of extraction buffer consisting of 1.4% vinylpyridine (v/v) to the supernatant and incubating at 65°C for 20 min. Then centrifuge at 10,000 rpm for 10 min and transfer 60 μl of the supernatant into a new tube. Add 240 μl of pre-cold acetone (-20°C) into the supernatant to a final concentration of 80% (v/v). Keep the samples at -20°C freezer for 1–2 hours or overnight, and then centrifuge at 10,000 rpm for 10 min and dried at room temperature. The final pellet was put into -20°C freezer for further use.

#### 2.2.2 Sample preparation

Add 60 μl of 50% acetonitrile (ACN) and 0.05% trifluoroacetic acid (TFA) to dissolve the precipitation for 1 hour at room temperature. Sinapinic acid (SA) was used as matrix, which was dissolved in 50% ACN and 0.05% TFA (10 mg/ml). Sample was spotted onto a MALDI-TOF Voyager DE Pro 100 sample size plate by 0.7 μl SA: 0.7 μl sample: 0.7 μl SA. The sample plate was air dried before analysis by MALDI-TOF-MS.

### 2.3 MALDI-TOF-MS

Biosystems Voyager DE Pro MALDI-TOF mass spectrometer was used in this study with delayed extraction technology operating. The mass spectrometer was operated in linear mode. The optimised instrument settings were as follows: 25kv acceleration voltage, 0.15% guide wire voltage and 94% plate voltage, 900 ns delay time in the mass weight range from 10 kDa to 50 kDa. Laser power was set from 1,800 minimum to 2,100 maximum. The final mass spectra recorded were the sum of 500 laser shots. All the samples were automatically accumulated in a random pattern over the sample area to provide the final spectra.

## Results

### 3.1 Optimization of Samples Extraction and MALDI-TOF-MS Settings

#### 3.1.1 Sample optimization for MALDI-TOF-MS analysis

Since some gliadins have similar molecular weight as LMW-GS, it usually interferes with the detection of LMW-GS especially when a high sensitive instrument such as MADI-TOF is used. It is essential to eliminate gliadins together with albumins and globulins to ensure a reliable result. In the optimized sample extraction method (described in 2.2.1. Protein extraction), 150 μl of 55% iso-propanol was added into the sample tube to displace the 150 μl extraction buffer at 65°C for 30 min. After centrifugation, the supernatant was analyzed by MALDI-TOF-MS and no peaks were appeared. This indicates that the non-glutenin proteins were eliminated completely from the pellet based on the optimized protein extraction method which is suitable for MALDI-TOF-MS analysis.

Vinylpyridine effects in LMW-GS extraction was also investigated. By adding vinylpyridine, the peak resolution reacted differentially over different molecular weight regions. It caused a slight reduction of peak separating resolution in the 30–34 kDa region. However, in the region above 34 KDa, the 1.4% vinylpyridine (v/v) addition significantly enhanced the resolution and reproducibility of the LMW-GS profiling.

Sample concentration is also one of the main factors for MALDI-TOF-MS analysis. Too high or too low sample concentration will cause some peaks to disappear. A range of sample preparation factors affect the final sample concentration, including dissolving time length, types of matrix solutions, the compositions and ratios of solvents, the resolving times, and final sample volumes. The tested volume of TFA varied from 0.1% to 3.0%, and ACN from 0 to 50.0%. After the best TFA, ACN and H2O composition was chosen based on the MALDI-TOF-MS spectra results, the samples dissolving times of 30 min, 1 h, 2 h, 3 h, 4 h and overnight were compared. Five sample dissolving volumes, 30, 60, 100, 200, 300 μL, were also compared. The final sample concentration was set by using 60 μl of 50% acetonitrile (ACN) and 0.05% trifluoroacetic acid (TFA) solution to dissolve the precipitation for 1 h at room temperature.

#### 3.1.2 Optimisation of MALDI-TOF-MS settings

MALDI-TOF instrumental setting is another factor that affects the profiling of protein mixtures. Different acceleration voltage (19, 20, 22, 25 kV), guide wire voltage (0.1%, 0.15%, 0.2%, 0.3%), plate voltage (90%, 91%, 92%, 93%, 94%), delay time (600, 700, 800, 900, 1000, 1100 ns), Laser power (1800–2800 maximum) and mass weight range were tested. The selected parameter combination (described in section 2.3. MALDI-TOF-MS) gave the best profiling results among all tested combinations.

### 3.2 Identification of LMW-GS Alleles

**Fig 1:**
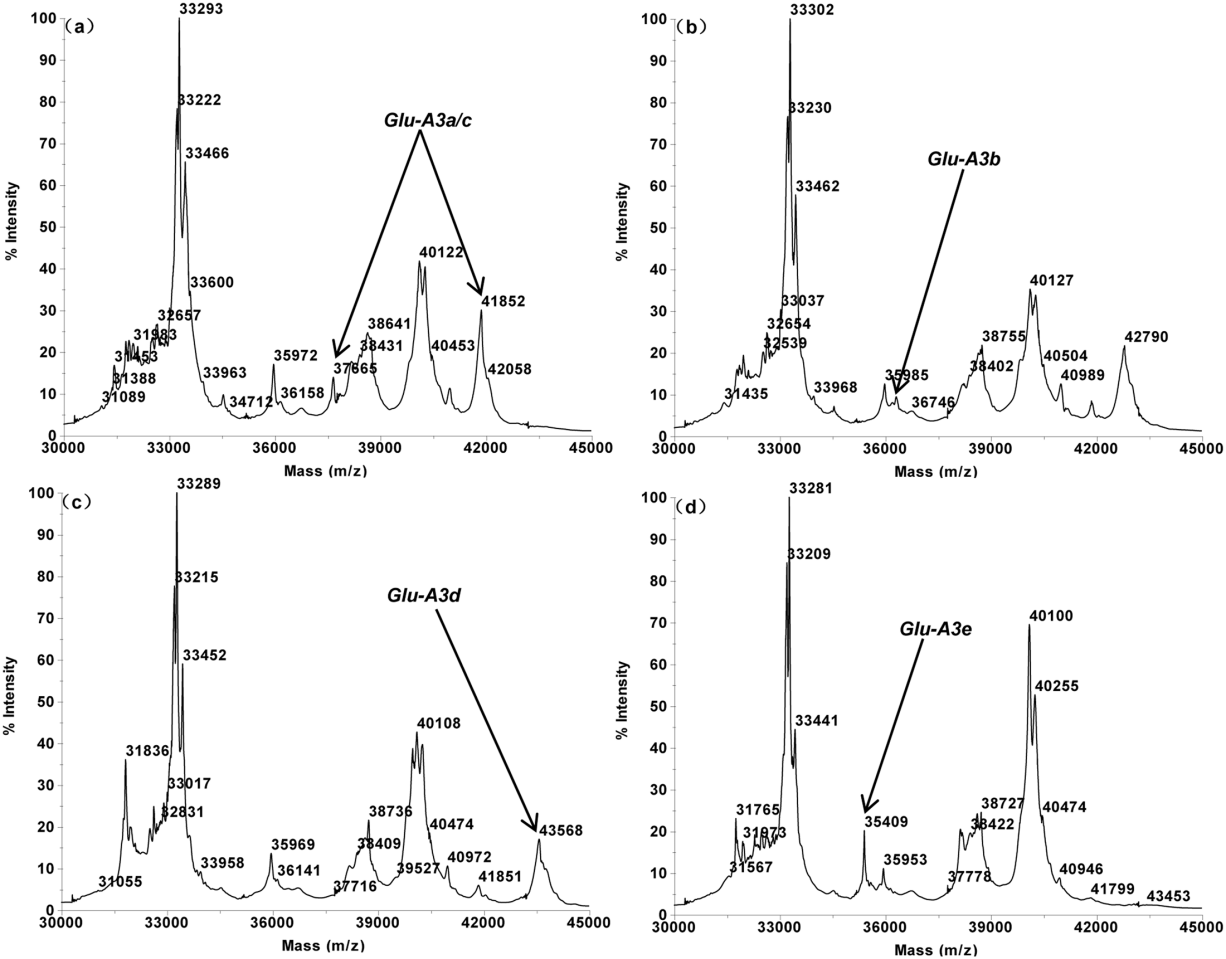
LMW-GS allele specific MALDI-TOF-MS spectrum patterns, arrows indicate identification allelic patterns of *Glu-A3*. a. *GluA3a/c* (Aroona), b. *Glu-A3b* (Aril 16–1), c. *Glu-A3d* (Aril 18–5), d. *Glu-A3e* (Aril 19–2).

**Fig 2:**
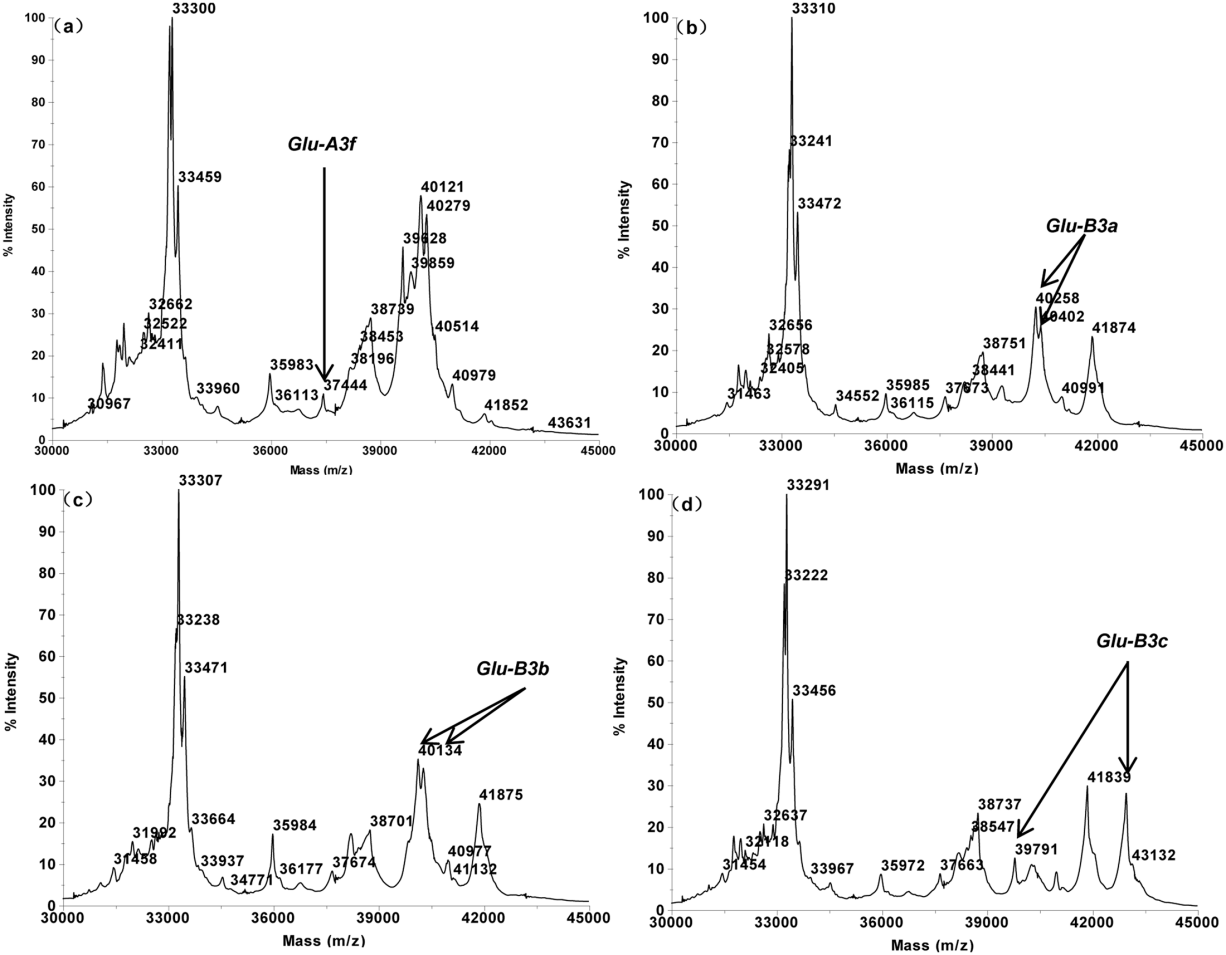
LMW-GS allele specific MALDI-TOF-MS spectrum patterns, arrows indicate identification patterns of *Glu-A3* and *Glu-B3*. a. *Glu-A3f* (Aril 20–1), b. *Glu-B3a* (Aril 21–2), c. *Glu-B3b* (Aroona), d. *Glu-B3c* (Aril 23–4).

**Fig 3:**
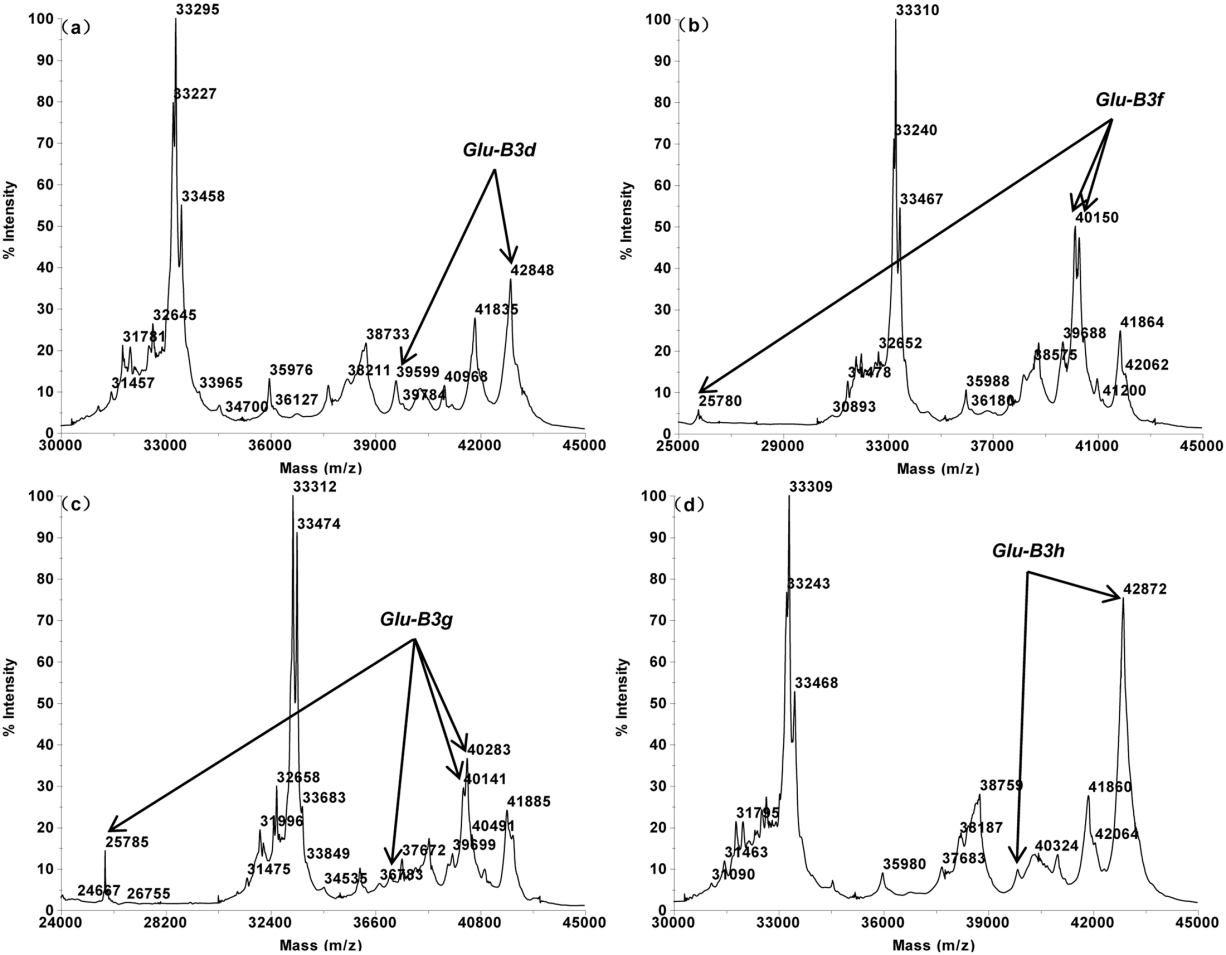
LMW-GS allele specific MALDI-TOF-MS spectrum patterns, arrows indicate identification patterns of *Glu-B3*. a. *GluB3d* (Aril 24–3), b *Glu-B3f* (Aril 26–1), c. *Glu-B3g* (Aril 27–6), d. *Glu-B3h* (Aril 28–4).

**Fig 4:**
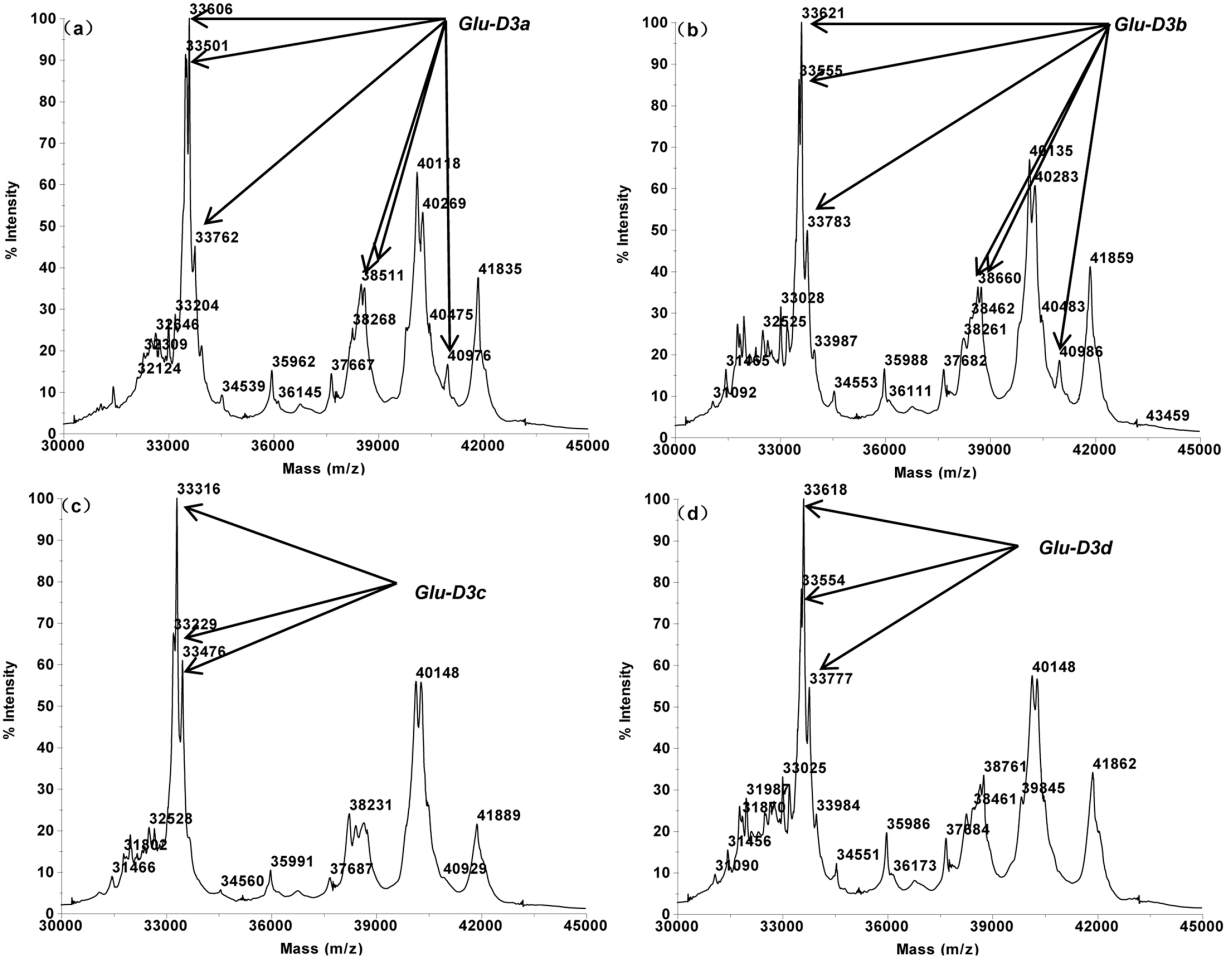
LMW-GS allele specific MALDI-TOF-MS spectrum patterns, arrows indicate identification patterns of Glu-D3. a. *Glu-D3a* (Aril 30–1) b. *Glu-D3b* (Aril 36–2), c. *Glu-D3c* (Aroona), d. *Glu-D3d* (Aril 33–1).

**Fig 5:**
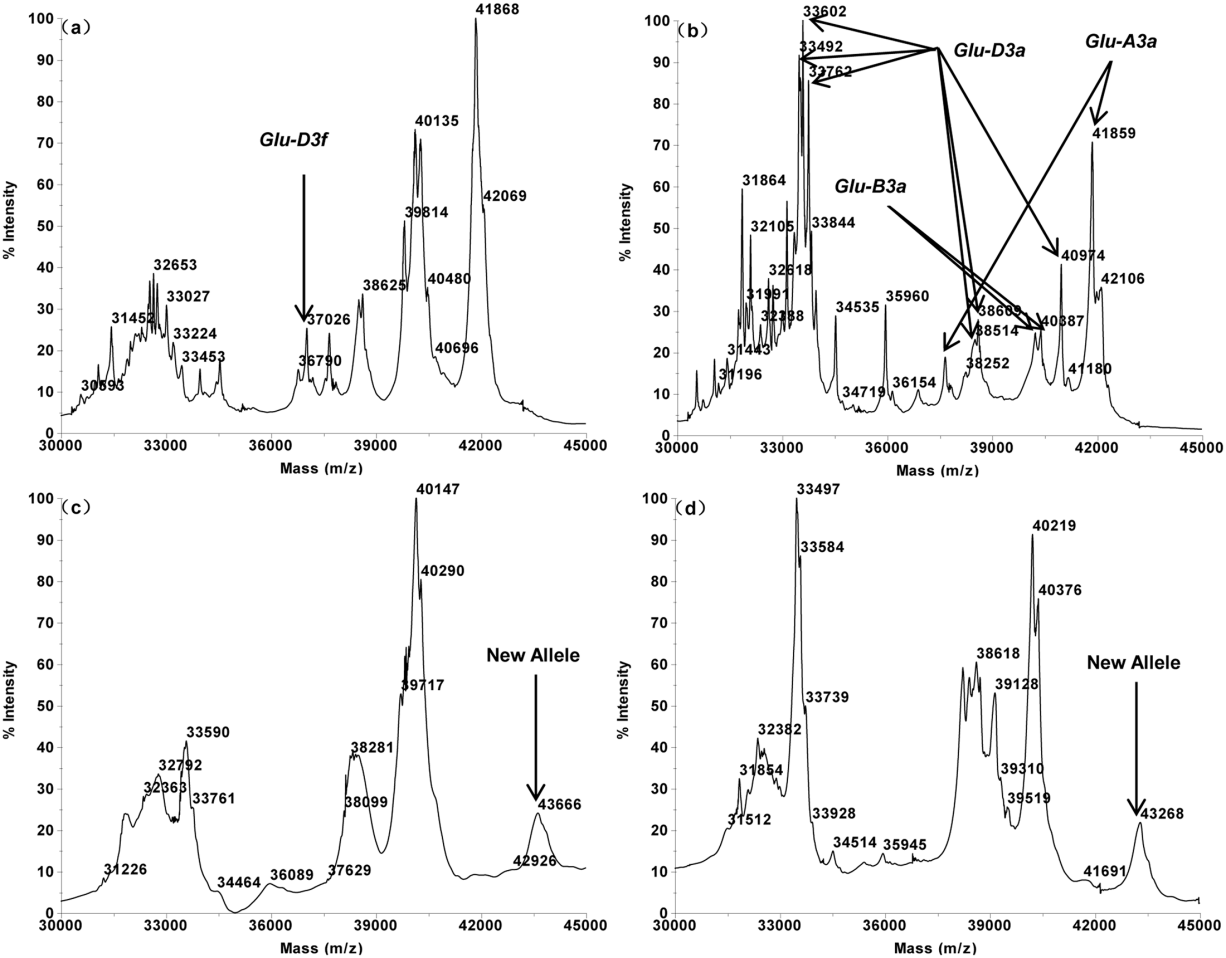
LMW-GS allele specific MALDI-TOF-MS spectrum patterns, arrows indicate identification patterns of *Glu-D3* and novel alleles. a. *Glu-D3f* (Aril 35–1), b. *Glu-A3a*, *B3a*, *D3a* (Chinese Spring), c. *Glu-B3b*, *D3d*, *A3*-43,666 Da (Baipimai), d. *Glu-B3a*, *D3d*, *A3*-43,268 Da (Hongmangmai).

The MALDI-TOF profiles of the 16 single Glu-3 substitution lines of Aroona (Table 1, S1 File) were initially used to establish a suite of characteristic protein peak combinations for all alleles. These allele specific spectrum peak patterns were then tested and verified by 25 hexaploid gene deletant lines (Table 2, S2 File) and 18 reference varieties from three countries (Table 3, S3 File). As a result, characteristic spectrum peak patterns were obtained for 17 LMW-GS alleles. These include 5 (b, a or c, d, e, f), 7 (a, b, c, d or i, f, g, h) and 5 (a, b, c, d, f) alleles at Glu-A3, Glu-B3 and Glu-D3 loci, respectively (Figs 1–5).

**Table 2:** Single *Glu-3* allele lines of *Triticum aestivum*.

Identifier	Line ID	*Glu-A3*	*Glu-B3*	*Glu-D3*
1	300/98	*a*	-	-
2	537/96	*b*	-	-
3	317/98	*b*	-	-
4	315/98	*c*	-	-
5	165/98	*d*	-	-
6	294/98	*f*	-	-
7	307/98	*e*	-	-
8	309/98	-	*a*	-
9	316/98	-	*b*	-
10	308/98	-	*c*	-
11	296/98	-	*c*	-
12	294/96	-	*d*	-
13	216/98	-	*f*	-
14	312/98	-	*g*	-
15	303/98	-	*h*	-
16	173/98	-	-	*a*
17	174/98	-	-	*a*
18	292/98	-	-	*b*
19	290/98	-	-	*c*
20	534/96	-	-	*c*
21	288/96.1	-	-	*d*
22	288/96.2	-	-	*d*
23	288/96.3	-	-	*d*
24	289/98.1	-	-	*f*
25	289/96.2	-	-	*f*

Data Explorer Raw Data files for all lines in table 2 are included in S2 File.

**Table 3:** Identification of LMW-GS alleles composition at loci *Glu-A3*, *Glu-B3*, *Glu-D3* in common wheat.

	Cultivar	*Glu-A3*	*Glu-B3*	*Glu-D3*	Origin
1	Westonia	*c/a or c*	*h/h*	*c/c*	CIMMYT
2	Halberd	*e/e*	*c/c*	*c/c*	CIMMYT
3	Tasman	*b/b*	*d/d or i*	*a/a*	CIMMYT
4	Trident	*e/e*	*h/h*	*c/c*	CIMMYT
5	Marquis	*e/e*	*b/b*	*a/a*	CIMMYT
6	Stiletto	*c/a or c*	*h/h*	*c/c*	CIMMYT
7	Carnamah	*c/a or c*	*d/d or i*	*c/c*	CIMMYT
8	Chinese Spring	*a/a or c*	*a/a*	*a/a*	France
9	Magdalena	*d/d*	*b/b*	*a/a*	France
10	Gabo	*b/b*	*b/b*	*b/b*	France
11	Cappelle-Desprez	*d/d*	*g/g*	*c/c*	France
12	Festin	*ef/f*	*b/b*	*c/c*	France
13	Insignia	*e/e*	*c/c*	*c/c*	France
14	Orca	*d/d*	*d/d or i*	*c/c*	France
15	Petrel	*d/d*	*h/h*	*c/c*	France
16	Thesee	*a/a or c*	*g/g*	*c/c*	France
17	Millewa	*c/a or c*	*g/g*	*b / b*	CIMMYT
18	Katepwa	*e/e*	*h/h*	*c/c*	Canada

Data preceding and following ‘‘/” are results by SDS-PAGE [21] and MALDI-TOF-MS, respectively.

Data Explorer Raw Data files for all lines in table 3 are included in S3 File.

Glu-A3 allele is typically composed by 1 or two peaks each, and is the simplest among the Glu-3 loci. The characteristic spectrum peak patterns of Glu-A3 alleles are: 36,320 Da for Glu-A3b, 37,665 + 41,852 Da for Glu-A3c, 43,568 Da for Glu-A3d, 35,409 Da for Glu-A3e, 37,444 Da for Glu-A3f (Figs 1 and 2a).

About 2–4 characteristic peaks existed in each Glu-B3 allele specific spectrum peak pattern: 40,258 + 40,402 Da for Glu-B3a, 40,134 + 40,287 Da for Glu-B3b, 39,791 + 42,949 Da for Glu-B3c, 39,599 + 42,848 Da for Glu-B3d, 25,780 + 40,150 + 40,301 Da for Glu-B3f, 25,785 + 37,221 + 40,141 + 40,283 Da for Glu-B3g, and 39,854 + 42,872 Da for Glu-B3h (Figs 2b, 2c, 2d and 3). It is noted that Glu-B3f is similar to Glu-B3g with the latter having an extra 37,221 Da peak.

The Glu-D3 alleles were found to be the most complicated among the 3 LMW-GS loci. Their characteristic peak number for each allele ranges from 1 to 6. Some characteristic peaks were clustered together and most alleles contained more than one clustered peak groups. In details, (33,501 + 33,606 + 33,762) + 38,511 + 38,605 + 40,976 Da matched Glu-D3a allele, (33,555 + 33,621 + 33,783) + 38,660 + 38,756 + 40,986 Da for Glu-D3b, (33,229 + 33,316 + 33,476) Da for Glu-D3c, and (33,554 + 33,618 + 33,777) Da for Glu-D3d (Fig 4). Glu-D3f allele was found to contain only one characteristic peak, 37,026 Da (Fig 5a).

Alleles Glu-A3a and Glu-A3c could not be differentiated by MALDI-TOF-MS due to their identical molecular masses; for the same reason, Glu-B3d and Glu-B3i were also difficult to differentiate. For this reason, Glu-A3a and Glu-A3c were assigned with the same spectrum peak pattern. Similarly, Glu-B3d and Glu-B3i also share the same characteristic pattern.

Some alleles possessed similar spectrum peak patterns, such as three Glu-B3 alleles (Glu-B3c, Glu-B3d and Glu-B3h), and two Glu-D3 alleles (Glu-D3a and Glu-D3b). However, the unique MALDI-TOF spectrum peak appearances and molecular weight combinations made these alleles easily differentiable. For example, Glu-B3c (39,791 + 42,949 Da) and Glu-B3d (39,599 + 42,848 Da) were highly similar, but the two peaks of Glu-B3c were of 192 Da and 101 Da higher than the two corresponding peaks of Glu-B3d. Practically, this makes it rather simple to differentiate these two alleles. Furthermore, the reproducible nature of the MALDI-TOF spectra also made the allele differentiation a straightforward task.

The LMW-GS compositions of the eighteen reference wheat cultivars identified by MALDI-TOF-MS are listed in Table 3. For most cultivars, the MALDI-TOF results were consistent with the SDS-PAGE results [21]. The only exception was exist in cultivar Festin at the Glu-A3 locus. Among these cultivars, allele Glu-A3c could not be differentiated from Glu-A3a, while Glu-B3d could not be distinguished from Glu-B3i by MALDI-TOF-MS. It is worth noting that Zhang et al. [24] also reported the difficulties in differentiating Glu-B3d and Glu-B3i due to nearly identical SDS-Page banding patterns of the two alleles. The results confirmed the feasibility of using MALDI-TOF-MS to analyze the compositions of LMW-GS.

**Fig 6:**
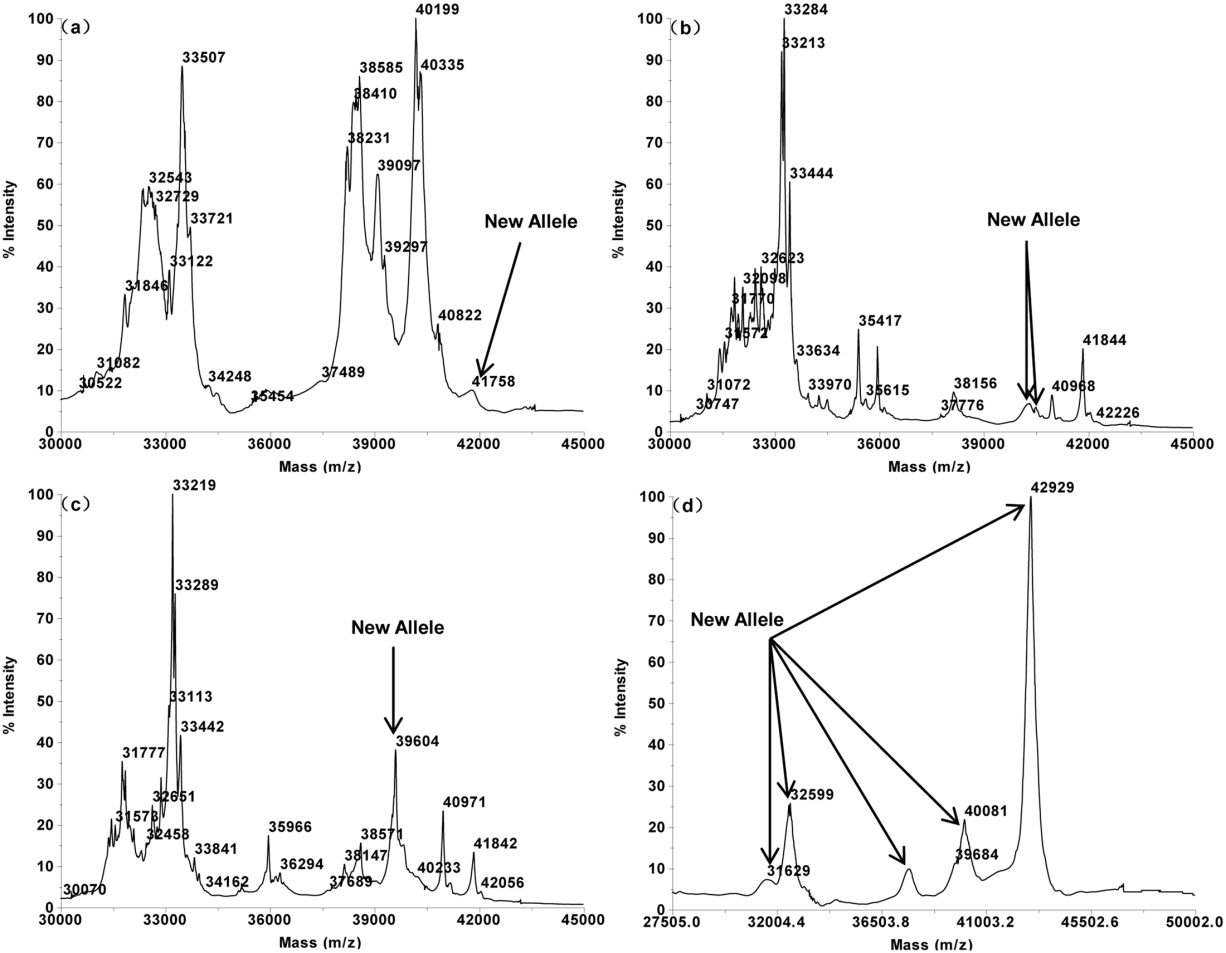
LMW-GS allele specific MALDI-TOF-MS spectrum patterns, arrows indicate identification patterns of novel alleles. a. *Glu-B3b*, *D3d*, *A3*-41,758Da (Hongmaoqiu), b. *GluA3e*, *D3c*, *B3*-(40,294 + 40,499) Da, (Hongkechong), c. *GluA3b*, *D3c*, B3-39,604 Da (Congqiumai), d. (31,629 + 32,599 + 37,657 + 40,081 + 42,929) Da (Yumai).

The MALDI-TOF results of the LMW-GS compositions for some accessions are shown in Figs 5b, 5c, 5d and 6.

### 3.3 Analysis LMW-GS Alleles in Wheat Cultivars and Breeding Lines

Two hundred and two lines of hexaploid wheat (including cultivars and some advanced breeding lines) were analyzed by MALDI-TOF-MS and the allele compositions of LMWGS obtained from the above allele specific spectrum peak pattern are listed in Table 4. Results revealed a total of 48 allele combinations among the studied genotypes and a total of 24 allelic variants at the three Glu-3 loci, and are shown in Table 5.

**Table 4:** Identification of LMW-GS alleles composition in common wheat.

	Accessions	*Glu-A3*	*Glu-B3*	*Glu-D3*
1	00RBC1548-50	*b*	*h*	*d*
2	00RBC1552-236	*a or c*	*h*	*c*
3	00RBC1736-29	*a or c*	*h*	*c*
4	00RBC1737-53	*a or c*	*d or i*	*c*
5	00w057-BE1-9	*d*	*f*	*a*
6	01RBC2035-2-G09	*a or c*	*h*	*c*
7	01RBC2078-43	*a or c*	*h*	*c*
8	01W666S-10-VB-1	*a or c*	*h*	*b*
9	02RBC2571-769	*a or c*	*h*	*c*
10	02RBC2584-14631-7	*d*	*h*	*a*
11	02RBC2686-167	*a or c*	*h*	*d*
12	02RBCgrp2-s15	*d*	*h*	*a*
13	02RBCgrp2-s151	*d*	*d or i*	*a*
14	02RBCgrp-s68	*b*	*b*	*a*
15	02W053-D04-011	*d*	*h*	*b*
16	02W287-D1-032	*a or c*	*b*	*c*
17	04IBWSN-042	*a or c*	*h*	*a*
18	04IBWSN-078	*a or c*	*g*	*a*
19	04IBWSN-113	*a or c*	*f*	*a*
20	04W172-16	*a or c*	*h*	*b*
21	04W172-25	*a or c*	*b*	*d*
22	34IBWSN301	*a or c*	*g*	*a*
23	98W816-D03-252	*a or c*	*b*	*c*
24	98W816-D09-556	*b*	*b*	*c*
25	98Y215-D3-17	*a or c*	*b*	*c*
26	98Y215-D3-18	*a or c*	*b*	*c*
27	99RBC1414-420	*a or c*	*h*	*c*
28	99W806-D3-418	*b*	*b*	*c*
29	99W817-NW50	*a or c*	*d or i*	*c*
30	Alta Blance	*b*	*h*	*b*
31	Alturas	*d*	*g*	*a*
32	AUS30666	*e*	*d or i*	*c*
33	AUS33519	*a or c*	*h*	*a*
34	AUS33558	*b*	*b*	*a*
35	BinnuVPM	*a or c*	*b*	*a*
36	CobDonor36	*b*	*h*	*a*
37	CobHR189	*a or c*	*h*	*c*
38	CobHR454	*b*	*b*	*b*
39	CobX2093selLrr	*a or c*	*g*	*b*
40	CobX2144	*a or c*	*f*	*c*
41	Derrimut	*b*	*b*	*d*
42	DM02.3*A31	*b*	*h*	*d*
43	DM02.3*D007	*b*	*h*	*a*
44	DM02.4*C15	*b*	*b*	*d*
45	DM02.4*D501	*b*	*b*	*d*
46	DM2002-1	*b*	*b*	*b*
47	DM2002-3	*b*	*b*	*b*
48	DM2002-CH157	*e*	*c*	*b*
49	DPI-Vic-PHS-024	*a or c*	*h*	*b*
50	DPI-Vic-PHS-025	*b*	*h*	*c*
51	DPI-Vic-PHS-030	*a or c*	*d or i*	*a*
52	Dundas31-2000	*a or c*	*g*	*c*
53	EGAWylie	*b*	*h*	*a*
54	GBA03.11.26	*a or c*	*h*	*a*
55	Gladius	*a or c*	*b*	*b*
56	Guardian	*b*	*b*	*b*
57	Baipimai	43,666Da	*b*	*d*
58	Hongmaoqiu	41,758 Da	*b*	*d*
59	Hongmangmai	43,268 Da	*a*	*d*
60	Yumai	42,929 Da, 40,081 Da, 37,675 Da, 32,599 Da and 31,629 Da	?	?
61	Hongkechong	*e*	40,294 + 40,499 Da	*c*
62	IDO624	*d*	*h*	*a*
63	IDO630	*d*	*g*	*a*
64	IDO636	*d*	*h*	*a*
65	Congqiumai	*b*	39,604 Da	*c*
66	KRL35	*f*	*b*	*a*
67	Lochsa	*a or c*	*h*	*b*
68	Peake	*b*	*b*	*b*
69	RAC1192lowPPOseln	*f*	*b*	*b*
70	RAC1263	*d*	*b*	*b*
71	RAC1423	*d*	*b*	*b*
72	RAC875.cascDH34	*a or c*	*b*	*b*
73	RAC875.CascDH80	*a or c*	*b*	*a*
74	RAC875.Unk24DH122	*a or c*	*b*	*b*
75	RAC875.Unk24DH180	*a or c*	*b*	*b*
76	RAC970	*e*	*h*	*c*
77	SUN325C	*b*	*b*	*b*
78	Thelin	*d*	*b*	*a*
79	Ventura	*b*	*h*	*a*
80	VR1128	*b*	*h*	*b*
81	VS0039	*b*	*h*	*c*
82	WAWHT2586	*b*	*b*	*a*
83	WAWHT2589	*b*	*b*	*a*
84	WAWHT2973	*a or c*	*b*	*c*
85	WAWHT2974	*a or c*	*b*	*c*
86	WAWHT2975	*a or c*	*b*	*c*
87	WAWHT2976	*a or c*	*b*	*c*
88	WAWHT2977	*a or c*	*b*	*c*
89	WAWHT2978	*a or c*	*h*	*c*
90	WAWHT2980	*a or c*	*h*	*b*
91	WAWHT2981	*a or c*	*h*	*b*
92	WAWHT2982	*a or c*	*h*	*a*
93	WAWHT2983	*a or c*	*b*	*b*
94	WAWHT2984	*a or c*	*d or i*	*b*
95	WAWHT2988	*b*	*f*	*a*
96	WAWHT2991	*b*	*f*	*a*
97	WAWHT2992	*b*	*f*	*a*
98	WAWHT2994	*b*	*f*	*a*
99	WAWHT2995	*b*	*f*	*a*
100	WAWHT2996	*b*	*g*	*a*
101	WAWHT2997	*b*	*g*	*a*
102	WAWHT3001	*b*	*b*	*b*
103	WAWHT3006	*b*	*d or i*	*a*
104	WAWHT3013	*f*	*h*	*b*
105	WAWHT3016	*a or c*	*h*	*a*
106	WAWHT3017	*a or c*	*h*	*b*
107	WAWHT3018	*a or c*	*h*	*b*
108	WAWHT3019	*a or c*	*h*	*b*
109	WAWHT3026	*a or c*	*h*	*a*
110	WAWHT3032	*b*	*b*	*a*
111	WAWHT3033	*b*	*a*	*b*
112	WAWHT3034	*b*	*b*	*b*
113	WAWHT3035	*b*	*b*	*b*
114	WAWHT3036	*b*	*b*	*b*
115	WAWHT3037	*b*	*b*	*b*
116	WAWHT3038	*a or c*	*b*	*c*
117	WAWHT3039	*b*	*h*	*b*
118	WAWHT3040	*b*	*b*	*a*
119	WAWHT3041	*b*	*b*	*b*
120	WAWHT3042	*b*	*b*	*a*
121	WAWHT3043	*b*	*b*	*b*
122	WAWHT3044	*b*	*b*	*a*
123	WAWHT3045	*b*	*b*	*b*
124	WAWHT3046	*b*	*b*	*b*
125	WAWHT3047	*b*	*b*	*b*
126	WAWHT3048	*b*	*b*	*b*
127	WAWHT3049	*b*	*b*	*a*
128	WAWHT3050	*b*	*b*	*b*
129	WAWHT3051	*b*	*b*	*a*
130	WAWHT3052	*b*	*b*	*b*
131	WAWHT3053	*b*	*b*	*b*
132	WAWHT3054	*b*	*b*	*b*
133	WAWHT3055	*b*	*b*	*b*
134	WAWHT3056	*b*	*b*	*b*
135	WAWHT3057	*b*	*b*	*b*
136	WAWHT3060	*f*	*h*	*c*
137	WAWHT3061	*a or c*	*h*	*c*
138	WAWHT3062	*a or c*	*b*	*c*
139	WAWHT3063	*a or c*	*b*	*c*
140	WAWHT3064	*a or c*	*b*	*c*
141	WAWHT3065	*a or c*	*h*	*c*
142	WAWHT3066	*a or c*	*h*	*d*
143	WAWHT3067	*b*	*h*	*c*
144	WAWHT3068	*b*	*h*	*c*
145	WAWHT3069	*b*	*b*	*b*
146	WAWHT3070	*b*	*h*	*c*
147	WAWHT3071	*a or c*	*b*	*a*
148	WAWHT3072	*a or c*	*h*	*c*
149	WAWHT3073	*a or c*	*h*	*a*
150	WAWHT3074	*a or c*	*h*	*b*
151	WAWHT3075	*a or c*	*h*	*b*
152	WAWHT3076	*a or c*	*h*	*c*
153	WAWHT3077	*a or c*	*h*	*c*
154	WAWHT3078	*a or c*	*h*	*c*
155	WAWHT3079	*a or c*	*g*	*a*
156	WAWHT3080	*a or c*	*g*	*a*
157	WAWHT3081	*d*	*b*	*b*
158	WAWHT3082	*b*	*b*	*a*
159	WAWHT3083	*b*	*b*	*a*
160	WAWHT3084	*b*	*b*	*b*
161	WAWHT3085	*a or c*	*h*	*b*
162	WAWHT3086	*a or c*	*h*	*b*
163	WAWHT3087	*a or c*	*b*	*c*
164	WAWHT3088	*f*	*h*	*b*
165	WAWHT3089	*a or c*	*b*	*c*
166	WAWHT3090	*b*	*b*	*b*
167	WAWHT3091	*b*	*b*	*b*
168	WAWHT3092	*a or c*	*b*	*b*
169	WAWHT3093	*a or c*	*h*	*b*
170	WAWHT3094	*a or c*	*h*	*c*
171	WAWHT3095	*a or c*	*h*	*b*
172	WAWHT3096	*a or c*	*h*	*b*
173	WAWHT3097	*a or c*	*h*	*b*
174	WAWHT3098	*d*	*h*	*a*
175	WAWHT3099	*a or c*	*h*	*a*
176	WAWHT3100	*a or c*	*h*	*b*
177	WAWHT3101	*b*	*b*	*b*
178	WAWHT3102	*a or c*	*h*	*b*
179	WAWHT3103	*a or c*	*h*	*c*
180	WAWHT3104	*b*	*b*	*b*
181	WAWHT3105	*a or c*	*h*	*b*
182	WAWHT3106	*a or c*	*h*	*b*
183	WAWHT3107	*a or c*	*h*	*b*
184	WAWHT3108	*a or c*	*g*	*b*
185	WAWHT3109	*a or c*	*h*	*b*
186	WAWHT3110	*a or c*	*b*	*b*
187	WAWHT3111	*b*	*b*	*b*
188	WAWHT3112	*b*	*b*	*b*
189	WAWHT3113	*a or c*	*h*	*b*
190	WAWHT3114	*a or c*	*h*	*b*
191	WAWHT3115	*a or c*	*h*	*a*
192	WAWHT3116	*a or c*	*b*	*a*
193	WAWHT3117	*a or c*	*h*	*b*
194	WAWHT3118	*a or c*	*h*	*a*
195	WAWHT3119	*a or c*	*h*	*a*
196	WAWHT3120	*a or c*	*b*	*a*
197	WAWHT3121	*a or c*	*b*	*a*
198	WAWHT3122	*a or c*	*h*	*a*
199	Wyalkatchm	*a or c*	*h*	*b*
200	Young	*a or c*	*h*	*b*
201	Young noVPM	*a or c*	*h*	*b*
202	ZWE45-039	*a or c*	*g*	*a*

**Table 5:** Allele combinations and variants at the three *Glu-3* loci in common wheat.

	*Glu-A3*	*Glu-B3*	*Glu-D3*	Varieties	Frequency (%)
1	*b*	*h*	*d*	2	0.99
2	*a or c*	*h*	*c*	16	7.92
3	*a or c*	*d or i*	*c*	2	0.99
4	*d*	*f*	*a*	1	0.50
5	*a or c*	*h*	*b*	29	14.35
6	*d*	*h*	*a*	5	2.48
7	*a or c*	*h*	*d*	2	0.99
8	*d*	*d or i*	*a*	1	0.50
9	*b*	*b*	*a*	12	5.94
10	*d*	*h*	*b*	1	0.50
11	*a or c*	*b*	*c*	15	7.43
12	*a or c*	*h*	*a*	12	5.94
13	*a or c*	*g*	*a*	5	2.48
14	*a or c*	*f*	*a*	1	0.50
15	*a or c*	*b*	*d*	1	0.50
16	*b*	*b*	*c*	2	0.99
17	*b*	*h*	*b*	3	1.49
18	*d*	*g*	*a*	2	0.99
19	*e*	*d or i*	*c*	1	0.50
20	*a or c*	*b*	*a*	6	2.97
21	*b*	*h*	*a*	4	1.98
22	*b*	*b*	*b*	32	15.84
23	*a or c*	*g*	*b*	2	0.99
24	*a or c*	*f*	*c*	1	0.50
25	*b*	*b*	*d*	3	1.49
26	*e*	*c*	*b*	1	0.50
27	*b*	*h*	*c*	5	2.48
28	*a or c*	*d or i*	*a*	1	0.50
29	*a or c*	*g*	*c*	1	0.50
30	*a or c*	*b*	*b*	7	3.47
31	43,666Da	*b*	*d*	1	0.50
32	41,758 Da	*b*	*d*	1	0.50
33	43,268 Da	*a*	*d*	1	0.50
34	42,929 Da, 40,081 Da, 37,675 Da, 32,599 Da and 31,629 Da	?	?	1	0.50
35	*e*	40,294 + 40,499 Da	*c*	1	0.50
36	*b*	39,604 Da	*c*	1	0.50
37	*f*	*b*	*a*	1	0.50
38	*f*	*b*	*b*	1	0.50
39	*d*	*b*	*b*	3	1.49
40	*e*	*h*	*c*	1	0.50
41	*d*	*b*	*a*	1	0.50
42	*a or c*	*d or i*	*b*	1	0.50
43	*b*	*f*	*a*	5	2.48
44	*b*	*g*	*a*	2	0.99
45	*b*	*d or i*	*a*	1	0.50
46	*f*	*h*	*b*	2	0.99
47	*b*	*a*	*b*	1	0.50
48	*f*	*h*	*c*	1	0.50

At the Glu-A3 locus, eight different spectrum peak patterns were detected. Among these, five corresponded to the known alleles while three did not match any known alleles. Alleles Glu-A3a or c and Glu-A3b were present at frequencies of 50.5% and 36.1%, respectively. Alleles Glu-A3d (6.9%), Glu-A3e (2.0%) and Glu-A3f (2.5%) occurred at lower frequencies. The three new spectrum peak patterns at the Glu-A3 locus were detected from three Chinese wheat lines. Wheat line Baipimai possessed a spectrum peak with molecular weight 43,665 Da, which can be confidently treated as a new allele (Fig 5c). Chinese wheat landraces Hongmangmai and Hongmaoqiu contained another two abnormal peaks, 43,267 Da (Fig 5d) and 41,758 Da (Fig 6a), respectively, representing two new alleles.

At the Glu-B3 locus, nine LMW-GS spectrum peak patterns were detected including seven known allele specific patterns and two new patterns that did not match any known alleles. Two alleles, Glu-B3b and Glu-B3h were the most frequent, with frequencies of 42.6% and 41.1% among the accessions, respectively. Five allele specific patterns, Glu-B3a, c, d or i, f and g, were present at lower frequencies of 1.0%, 0.5%, 3.5%, 4.0% and 5.9%, respectively. For Chinese landrace lines Hongkechong (40,294 + 40,499 Da) and Congqiumai (39,603 Da), the speculative Glu-B3 peak patterns did not correspond to known alleles, indicating new Glu-B3 alleles (Fig 6b and 6c).

For the Glu-D3 locus, allele Glu-D3b was the most frequent one (41.1%), followed by Glu-D3a (29.7%) and Glu-D3c (23.3%). Eleven accessions possessed Glu-D3d at a frequency of 5.4%. It is worth noting that sample Yumai expressed only five subunits 42,929 Da, 40,081 Da, 37,675 Da, 32,599 Da and 31,629 Da that did not correspond to any known allele patterns, indicating three new LMW-GS alleles with one each on the three Glu-3 loci (Fig 6d).

## Discussion

The current methodology development involved optimization of sample extraction and instrument settings to generate reproducible diagnostic spectrum profiles for wheat LMW-GS. Based on MALDI-TOF settings and models, over 100 wheat samples can be readily analyzed for LMW-GS alleles, indicating a high throughput nature. A total of 17 known LMW-GS alleles were found with matching spectrum peak patterns, including 5 (b, a or c, d, e, f), 7 (a, b, c, d or i, f, g, h) and 5 (a, b, c, d, f) alleles for the Glu-A3, Glu-B3 and Glu-D3 loci, respectively. According to LMW-GS allele characteristic peak patterns, 48 LMW-GS allele combination or genotypes in common wheat (Triticum aestivum L.) were identified.

For the 18 reference cultivars, the spectrum scoring results of most cultivars are consistent with the SDS-PAGE results published previously excepting cultivar Festin, which was identified to contain the Glu-A3ef allele by Branlard et al. [25] but appeared as Glu-A3f allele in our study. As it was difficult to differentiate between Glu-A3e and Glu-A3f through SDS-PAGE, the two alleles were combined as Glu-A3ef previously [25]. However, our established MALDI-TOF procedure can clearly differentiate these two alleles.

Line Aril 15–4 (Glu-(A3a, B3b, D3c)) and Aroona (Glu-(A3c, B3b, D3c)) displayed the same MALDI-TOF spectra. The spectra of cv Chinese Spring, which is the Glu-A3a donor parent for Aril 15–4, were identical to these of Aril 15–4 and Aroona, all having the same characteristic peaks at the Glu-A3 locus (37,665 + 41,852 Da). For this reason, alleles Glu-A3a and Glu-A3c were not differentiable through MALDI-TOF. Aril 16–1 (Glu-(A3b, B3b, D3c)) and Aril 18–5 (Glu-(A3d, B3b, D3c)) both expressed one of the Glu-A3ac characteristic peak 41,852 Da but did not contain the 37,665 Da peak, thus the spectrum peak combination (37,665 + 41,852) Da were used to represent Glu-A3ac. Glu-A3d contained another two characteristic peaks, including 39,985 Da and 43,587 Da that is of the highest molecular weight among all LMW-GS spectra and only existed in Glu-A3d. This made it convenient to identify Glu-A3d by simply examining the existence of the maximal peak (43,587 Da); the Glu-A3f contained another two peaks 39,682 Da and 37,444 Da with the latter being exclusively existed in Glu-A3f. For this reason, peak 37,444 Da was adapted as a scoring mark for allele Glu-A3f. All of these were confirmed by the Aril lines, gene deletant lines, the reference cultivars, and most varieties being analyzed.

By using MALDI-TOF technology, we were able to identify the LMW-GS allelic compositions of 197 wheat lines out of 202. Three Chinese landrace lines expressed no characteristic peak patterns for known Glu-B3 alleles, suggesting novel Glu-B3 alleles in these wheat lines. No peak pattern could be identified to match with known alleles from landrace Yumai, indicating novel alleles at the three Glu-3 loci. The characteristic peaks for Glu-D3c were (33,229 + 33,316 + 33,476) Da, which were used as the core spectrum peak pattern and criteria in determining Glu-D3c allele. However, four types of sub-allele variates were found for Glu-D3c that each contains different set of additional peaks apart from the three core peaks, including (38,511 + 38,605) + 40,976 Da, (38,660 + 38,756) + 40,986 Da, 40,976 Da, (33,229 + 33,316 + 33,476) + (38,660 + 38,756) Da. This makes a total of 5 allelic types for the conventionally known Glu-D3c allele. This clearly demonstrates an enhanced power of MALDI-TOF procedure in analyzing LMW-GS allelic compositions. Such analytical power is desirable in modern wheat breeding since LMW-GS composition is highly polymorphic and high resolution identification of LMW-GS protein compositions is critical for efficiently utilizing the genetic variations [26–28].

It is worth reemphasizing that new Glu-3 alleles have been identified from our limited germplasm collection through the established MALDI-TOF procedure. The novel subunits associated with the abnormal spectrum peaks 43,665 Da, 43,267 Da, 41,758 Da may play a particular role in determining the viscoelastic properties of wheat dough. A more detailed study is required to characterize those novel alleles.

As a summary, a high efficient MALDI-TOF-MS procedure is established in the current study which is a rapid, simple, accurate and reliable method to identify wheat LMW-GS allele compositions. Through this approach, the complex LMW-GS can be readily differentiated. It can be used as an alternative approach for rapid identification of wheat LMW-GS in wheat breeding, which is most suitable for dealing with a large number of samples in a short period.

Disclaimer: This work is financially funded by Australian Grain Research & Development Corporation project: UMU00028. The funder had no role in study design, data collection and analysis, decision to publish, or preparation of the manuscript.

## Supporting Information

S1 File (Raw Data for Table 1) Mass spectrum files for each wheat lines listed in Table 1. The data is compressed and in Biosystems MALDI-TOF software Data-Explorer format.
(ZIP)

S2 File (Raw Data for Table 2) Mass spectrum files for each wheat lines listed in Table 2. The data is compressed and in Biosystems MALDI-TOF software Data-Explorer format.
(ZIP)

S3 File (Raw Data for Table 3) Mass spectrum files for each wheat lines listed in Table 3. The data is compressed and in Biosystems MALDI-TOF software Data-Explorer format.
(ZIP)
